# Artificial Intelligence Enabled Dielectric Fingerprinting of Arrhythmogenic Substrates: A Precision Paradigm for Tissue Characterization Guided Cardiac Electrophysiology

**DOI:** 10.3390/s26196090

**Published:** 2026-09-25

**Authors:** Lakshmi Sree Pugalenthi, Sidhartha Gautam Senapati, Divyanshi Sood, Areesh Fatima Sahito, Bargavi Kathirvel, Avni Arora, Kaaviyashri Saraboji, Anmolpreet Kaur, Simardeep Kaur Bumrah, Manjunath Krishnappa, Jieun Lee, Mohammed Naveed Shariff, Gayathri Yerrapragada, Poonguzhali Elangovan, Sancia Mary Jerold Wilson, Jayavinamika Jayapradhaban Kala, Shuvashis Dey, Dipankar Mitra, Shiva Sankari Karuppiah, Rashi Bilgaiyan, Suganti Shivaram, Scott A. Helgeson, Christopher V. DeSimone, Elena G. Tolkacheva, Shivaram P. Arunachalam

**Affiliations:** 1 Department of Internal Medicine, Mercy Catholic Medical Center, Philadelphia, PA 19143, USA; 2 Department of Internal Medicine, Texas Tech University Health Sciences Center, El Paso, TX 79905, USA; senapati.sidhartha36@gmail.com; 3 Department of Internal Medicine, UCHealth Parkview Medical Center, Pueblo, CO 81003, USA; 4 Digital Engineering & Artificial Intelligence Laboratory (DEAL), Mayo Clinic, Jacksonville, FL 32224, USAkaaviyasaraboji@gmail.com (K.S.); krishnappa.manjunath@mayo.edu (M.K.); rashi.bilgaiyan@gmail.com (R.B.);; 5 Department of Clinical Neuroscience, Virginia Tech, Blacksburg, VA 24061, USA; 6 Microwave Engineering & Imaging Laboratory (MEIL), Mayo Clinic, Jacksonville, FL 32224, USA; shuvashis.dey@ndsu.edu (S.D.); dmitra@uwlax.edu (D.M.); 7 Department of Electrical and Computer Engineering, North Dakota State University, Fargo, ND 58102, USA; 8 Department of Computer Science and Computer Engineering, University of Wisconsin, La Crosse, WI 54601, USA; 9 Division of Pulmonary Medicine, Department of Medicine, Mayo Clinic, Jacksonville, FL 32224, USA; 10 Department of Critical Care Medicine, Mayo Clinic, Jacksonville, FL 32224, USA; 11 Division of Heart Rhythm Services, Department of Cardiovascular Medicine, Mayo Clinic, Rochester, MN 55905, USA; 12 Department of Biomedical Engineering, University of Minnesota, Minneapolis, MN 55455, USA

**Keywords:** cardiac arrhythmias, dielectric properties, local impedance, myocardial tissue characterization, artificial intelligence, cardiac electrophysiology

## Abstract

Background: Electrical and dielectric properties of the myocardium depend on its composition, fibrosis, edema, ischemia, and heat damage, and hence may play an important role in defining the electrophysiologic substrate. Local impedance and other impedance-based methods give information about these parameters that is available to clinicians, but not direct or substitutable for intrinsic conductivity and permittivity of the tissue. The use of artificial intelligence has been thoroughly studied for the ECGs, electrograms, images, and computational data analysis, but not for dielectric or impedance measurements. Objective: The study aims to review the scientific basis of dielectric properties of heart muscle critically in cardiology and electrophysiology, to differentiate between existing clinical and conceptual applications of myocardial dielectrics, and to estimate current and potential contributions of artificial intelligence to their interpretation. Data Sources and Study Selection: The current work represents a structured narrative review in PubMed, Scopus, and Google Scholar databases from inception until May 2026 based on various combinations of keywords that refer to myocardial impedance, dielectric properties, conductivity, permittivity, local impedance, dielectric imaging, cardiac electrophysiology, catheter ablation, machine learning, and artificial intelligence. This review included experimental, ex vivo, animal, computational, and human studies related to myocardial dielectric properties and their use in electrophysiology. Evidence was classified according to four categories: direct human clinical evidence; preclinical/translation evidence; indirect evidence from related techniques; and extrapolation concept. As a result of heterogeneity between studies, sensing technologies, and outcomes, the results have been analyzed narratively. Key Findings: Evidence regarding the usage of AI algorithms, which have been primarily trained on myocardial dielectric or catheter-based local impedance data, is extremely scarce and mostly consists of preclinical or ex vivo data. On the contrary, the majority of clinically used AI algorithms in the field of electrophysiology make use of ECG data, intracardiac electrogram data, image processing, or computational modeling without any inputs related to dielectrics or local impedance. Conclusions and Implications for Practice: Dielectric sensing is an attractive supplement to traditional methods of voltage mapping and imaging, although the clinical readiness of dielectric sensing greatly varies depending on the application. At the present time, the existing data provide a basis for utilizing the local impedance measurement in the ablation procedure and evaluation of the myocardium properties, while the combination of artificial intelligence and dielectric sensing is still experimental. Before being introduced into the clinical electrophysiology practice, signal acquisition standardization and prospective multicenter trials are needed.

## 1. Introduction

Cardiac arrhythmias represent a heterogeneous spectrum of rhythm disorders that significantly contribute to cardiovascular morbidity and mortality worldwide. These conditions arise from abnormalities in cardiac electrical impulse generation or conduction, resulting in irregular heart rhythms. These range from relatively benign premature beats to fatal arrhythmias associated with hemodynamic instability and sudden cardiac death.

Cardiac arrhythmias are common worldwide and their prevalence increases significantly with age and the presence of structural heart disease. Arrhythmia is the most commonly sustained among atrial fibrillation (AF), affecting about 1–2% of the general population and more than 10% of individuals over 80 years old, with a lifetime risk of approximately one in three to four adults. Its global burden exceeds 60 million people and continues to rise due to aging populations and improved detection. A substantial body of evidence links AF to adverse clinical outcomes including heart failure and stroke. Large-scale population-based studies have shown that individuals with atrial fibrillation and associated atrioventricular conduction disorders experience substantially higher rates of cardiovascular events and all-cause mortality compared to individuals without arrhythmias, and those with AF demonstrated significantly increased risk of heart failure (hazard ratio ~3.99) and ischemic stroke (hazard ratio ~1.59) over long-term follow-up [1].

Ventricular arrhythmias, particularly ventricular tachycardia (VT) and ventricular fibrillation (VF), are major drivers of sudden cardiac death (SCD) in both structural heart disease and primary electrical disorders. These arrhythmias are often triggered by premature beats and sustained by complex reentrant circuits, highlighting their lethal potential across diverse clinical settings. Inherited channelopathies including long-QT syndrome, Brugada syndrome, and catecholaminergic polymorphic ventricular tachycardia (CPVT) further contribute to this burden, particularly in younger individuals without structural heart disease. For example, CPVT is associated with frequent syncopal episodes, bidirectional VT, and sudden cardiac arrest if left unrecognized and untreated, with mortality rates approaching 50% by age 35 in the absence of appropriate management [2].

Frequent premature ventricular complexes (PVCs) have also been associated with increased risks of incident atrial fibrillation, heart failure, ischemic stroke, and death, with evidence suggesting a burden-dependent relationship in which higher PVC frequency confers progressively greater risk of adverse outcomes [3].

Bradyarrhythmias also carry significant clinical implications. Sinus node dysfunction and advanced atrioventricular block become increasingly prevalent with aging and often require permanent pacemaker implantation. If untreated, severe bradyarrhythmias may result in syncope, worsening heart failure, or even sudden cardiac death; however, prognosis is generally favorable when timely pacing therapy is provided [4,5].

Overall, cardiac arrhythmias contribute substantially to hospital admissions, healthcare resource utilization, and deterioration in quality of life across diverse patient populations, including those with cardiogenic shock or ischemic heart disease [6].

The global burden of arrhythmias continues to rise, largely driven by population aging, the increasing prevalence of cardiovascular risk factors such as hypertension, diabetes, and obesity, and improved survival from cardiovascular disease.

Despite advances in cardiac electrophysiology, significant limitations remain in the diagnosis and treatment of arrhythmias. Conventional diagnostic modalities, including surface electrocardiography and short-term ambulatory monitoring, may fail to capture paroxysmal or asymptomatic arrhythmic episodes, leading to underdiagnosis or delayed intervention. Although prolonged rhythm monitoring, antiarrhythmic pharmacotherapy, catheter ablation, and implantable device therapies have enhanced treatment outcomes, these interventions remain limited by arrhythmia recurrence, procedural complications, proarrhythmic potential, and suboptimal risk stratification. Collectively, these limitations emphasize the need for more sensitive diagnostic frameworks and more precise, mechanism-targeted therapeutic approaches.

Surface electrocardiography (ECG) and short-duration Holter monitoring continue to serve as core modalities for arrhythmia detection. However, their diagnostic utility is inherently constrained by limited recording duration, which reduces sensitivity for intermittent or asymptomatic arrhythmias, including paroxysmal atrial fibrillation and non-sustained ventricular tachycardia (NSVT). Extended rhythm monitoring using implantable loop recorders (ILRs) improves detection rates for sporadic arrhythmic events compared to conventional 24 h Holter monitoring. Nevertheless, ILRs are limited by the need for invasive implantation, increased cost, and limited sensitivity for very brief arrhythmic episodes, restricting their widespread applicability. Furthermore, variability in device-specific detection algorithms, along with issues related to patient adherence and signal quality, may compromise arrhythmia classification, particularly for asymptomatic or short-duration rhythm disturbances [7,8].

Electroanatomic voltage mapping (EAM) and conventional imaging modalities including echocardiography, computed tomography (CT), and cardiac magnetic resonance (CMR) provide essential information on arrhythmogenic substrates to guide ablation planning. Pre-procedural multimodal imaging can facilitate the identification of scar and border zones and improve procedural strategies; however, limitations persist in spatial resolution, tissue characterization, and integration with mapping systems, especially in patients with implanted cardiac devices or extensive artifacts. The accuracy of substrate delineation using standard voltage thresholds may underestimate true arrhythmogenic tissue, and imaging artifacts or limited resolution can obscure critical functional corridors relevant to ventricular tachycardia circuits. These constraints result in incomplete substrate identification and may reduce the precision of ablation targeting [9,10].

Despite substantial advances in mapping technologies and catheter-based interventions, important procedural and prognostic challenges continue to limit long-term success in arrhythmia management. During electrophysiologic study for ventricular tachycardia (VT), the non-inducibility hinders precise circuit localization and targeted ablation planning. Although substrate-based mapping strategies were developed to overcome this limitation by identifying abnormal tissue even when arrhythmia is not inducible, these approaches remain constrained by heterogeneous myocardial architecture. In addition, the formation of non-transmural ablation lesions can leave residual viable myocardium capable of sustaining recurrent VT, even when the procedure appears successful.

Similar challenges are observed in atrial fibrillation (AF), where recurrence following pulmonary vein isolation remains frequent, reflecting complex substrate heterogeneity, incomplete lesion sets, and progressive structural remodeling. Beyond procedural factors, limitations in contemporary risk stratification models further complicate management decisions. Current tools demonstrate insufficient sensitivity and specificity for predicting sudden cardiac death, arrhythmia recurrence, or disease progression across diverse patient populations, thereby restricting their utility in guiding primary preventive strategies. Overall, these procedural and predictive constraints highlight a persistent gap between technological advancement and precise, individualized arrhythmia care, highlighting the need for more accurate substrate characterization and improved risk assessment methodologies [11].

Dielectric properties are clinically attractive because they reflect underlying structural and compositional changes within myocardial tissue. Pathological processes such as fibrosis, edema, scar formation, fatty infiltration, and ischemia alter intracellular–extracellular balance, membrane integrity, and water content, thereby influencing local electrical impedance and permittivity. These changes create measurable heterogeneity in dielectric signatures that correspond to arrhythmogenic substrates. For example, fibrotic or scarred myocardium typically demonstrates reduced conductivity and altered permittivity compared with healthy tissue, whereas edema and acute ischemia produce distinct dielectric patterns related to increased water content. This link between structural remodeling and impedance/permittivity heterogeneity provides a mechanistic basis for identifying abnormal tissue beyond voltage amplitude alone [12,13].

Experimental and human mapping studies further demonstrate that fibrotic and ischemic myocardium exhibit electrical characteristics distinct from normal tissue, forming the basis for the dielectric differentiation of arrhythmogenic substrate.

Tissue heterogeneity is central to arrhythmogenesis, as spatial dispersion of conduction and refractoriness promotes reentry and abnormal impulse propagation. Substrate mapping studies have shown that heterogeneous scar and border-zone tissue form the structural basis for ventricular tachycardia circuits, supporting techniques that better characterize tissue variability. Recent advances in catheter-based technologies enable real-time substrate characterization during electrophysiologic procedures, allowing tissue-level assessment beyond conventional voltage mapping. Feasibility studies suggest that enhanced substrate definition during ablation may improve procedural guidance and lesion targeting.

Dielectric-based assessment may complement electroanatomic voltage mapping and imaging modalities such as echocardiography, computed tomography, and cardiac magnetic resonance by providing direct tissue characterization. Multimodality integration has been shown to improve scar identification and substrate delineation in ventricular arrhythmia management. Improved discrimination between viable myocardium, dense scar, and heterogeneous border zones may enhance ablation planning, and substrate-based strategies guided by refined scar characterization have been associated with reduced arrhythmia recurrence in patients with structural heart disease [14,15,16,17].

This review offers a clinician-focused overview of how myocardial dielectric properties, together with emerging artificial intelligence (AI) approaches, may enhance the understanding, detection, and treatment of major cardiac arrhythmias. It focuses on translating biophysical tissue characteristics into clinically relevant insights by connecting electrophysiologic mechanisms with dielectric sensing technologies and AI-driven signal analysis used in modern cardiac mapping and imaging systems. For each major arrhythmia type including atrial fibrillation, atrial flutter and supraventricular tachycardias, ventricular tachyarrhythmias, and bradyarrhythmias, the discussion begins with the underlying pathophysiology and structural remodeling processes that generate arrhythmogenic substrates such as fibrosis, inflammation, ischemia, and conduction system degeneration [18].

The review then explores how these structural and cellular alterations alter myocardial dielectric properties, including conductivity, permittivity, and impedance, which reflect changes in tissue composition, cellular architecture, and extracellular matrix organization [12,19].

Finally, the potential contribution of AI-assisted analysis of dielectric signals is discussed, highlighting how machine-learning-based approaches may enhance arrhythmia detection, improve substrate mapping, guide catheter ablation strategies, and enable real-time tissue characterization within modern electrophysiology workflows [20].

## 2. Review Approach and Evidence Classification

This study provides a structured narrative review on myocardial dielectric properties, their biological background, techniques for the assessment, clinical use cases, and perspectives of their interpretation using artificial intelligence technology. A literature search in PubMed, Scopus, and Google Scholar databases was performed from the date of their creation till May 2026 using the following combined keywords: “myocardial impedance,” “local impedance,” “dielectric properties,” “dielectric imaging,” “conductivity,” “permittivity,” “cardiac electrophysiology,” “arrhythmogenic substrate,” “catheter ablation,” “artificial intelligence,” “machine learning,” and “digital twin.” The references cited in relevant papers were also analyzed.

Inclusion criteria included articles that assessed myocardial or intracardiac dielectric properties, impedance during electrophysiological interventions, biological background of dielectric properties of myocardium, or computational approaches potentially usable for interpretation of the dielectric signal. Due to the immature stage of development of the subject matter, evidence was taken from human clinical trials, animal studies, ex vivo tissue experiments, computational research, and from consensus documents and technical reports.

The literature search involved PubMed, Scopus, and Google Scholar from the earliest date available until May 2026, supplemented with reference list searching. Overall, 122 papers were considered for the narrative synthesis. In consideration of the fact that this is a structured narrative review and a prospective record of screening has not been kept, it is not possible to provide an accurate number of initial citations, duplicates eliminated, and full-text papers discarded. Papers were excluded if they pertained to noncardiac tissues, were not relevant to cardiac electrophysiology, did not consider dielectric or impedance-based measurement techniques, or offered inadequate original/clinical/technical details.

To separate evidence about technological feasibility from evidence of clinical applicability, it was divided into four categories: (1) direct clinical evidence—dielectric properties measured during human electrophysiology procedures or in patients’ cohort; (2) preclinical or translational evidence—animal, ex vivo, and experimental studies; (3) indirect evidence—application of artificial intelligence to electrograms, ECG, imaging, or other electrophysiological parameters but not to dielectric properties; (4) conceptual applications—application potentially based on biological or technological background but not studied directly.

Given the large heterogeneity between the methods used in terms of sensing frequency, catheter platform, tissue preparation, patient cohort, and findings, quantitative meta-analysis could not be performed. Therefore, the review focuses on the maturity and limitations of the evidence.

Since no validated bias assessment tool is available for this highly heterogeneous group of humans, animal, ex vivo, computational, consensus, and technical studies, any numerical grading of evidence was not attempted. Evidence assessment relied on study setting, directness, validation approach, sample size, prospective versus retrospective approach, multicentricity, and clinical relevance to electrophysiology.

To facilitate interpretation and avoid conflating technological feasibility with established clinical utility, Table 1 summarizes the maturity and source of evidence supporting the principal applications discussed in this review.

## 3. Biophysical Basis and Measurement of Myocardial Dielectric Properties

### 3.1. Core Dielectric Parameters and Myocardial Microstructure

The myocardial dielectric characteristics can be seen as a result of the interplay between electric fields and the components of the tissue of the heart itself. Conductivity, permittivity, resistivity, impedance, and phase angle are the main measurable characteristics. Sensing frequency influences those characteristics because the low frequency current travels predominantly along the extracellular pathway while the high-frequency current is involved in the processes inside the cells themselves [19,22,49].

The impact of fibrosis in arrhythmogenesis is determined by the degree of lost electrical connection among myocytes and the distribution pattern of the lost connections (Table 1). Normally, the cardiac action potential propagation is through gap junction channels [50]. While gap junctions cover the entire periphery of neonatal myocytes, cell polarization occurs over the postnatal maturation period, and large gap junctions are noticed at the intercalated disks to provide end-to-end connections, while smaller junctions provide side-to-side connections [51]. Transverse connections are dependent on the lateral extent of the muscle cells, a factor, which is determined by the collagenous septa. In Bachmann’s bundle, such septa separate myocytes by 1–2 mm. This sets cardiac electrical anisotropy according to tissue architecture and conduction velocity ratio ranges from unity in the atrial wall to between three and six in the highest alignment areas. On a microscopic level, conduction is saltatory, and the transverse pathway is naturally discontinuous with clearly discernible temporal delays between neighboring clusters of myocytes [52,53,54]. Longitudinal conduction is only mildly inhibited by a reduction in sodium currents, and uncoupling of the junctions allows slow but otherwise unblockable conduction determined predominantly by calcium currents. This discontinuous process is dependent on calcium to maintain the plateau phase long enough to bring the unstimulated strands up to their activation threshold [55,56].

On the microscopic level, passive electrical properties in the cardiac T-tubular system (TATS) are vital to signal uniformity. Using formal analogy diffusion and electrical conductivity, the network’s effective electrical conductivity was quantified using fluorescence recovery after photobleaching (FRAP) microscopy. This results in conduction defects and asynchrony often seen in diseased cardiac cells [57].

Physical barriers such as scar tissue or fibrosis have a predominant effect on increasing APD dispersion, because as physical insulators, they act to decouple the electrical syncytium in the heart [58]. Normally, the differences are filtered out by a “spatial filter” inherent in electrotonic coupling but when there is a barrier, cells on either side can manifest their ionic differences to the fullest. This situation results in steep gradients with one region remaining refractory while an adjacent region is fully recovered. The structural barriers increase the tortuosity and capacitive loading (source–sink mismatch), slowing conduction and favoring the reentrant circuits that sustain the lethal arrhythmias [58]. Replacement of viable myocardium by poorly conductive collagen and loss of gap–junction coupling reduce effective tissue conductivity and consequently increase impedance, particularly at lower sensing frequencies [59,60,61].

Figure 1 summarizes how dielectric properties are intertwined with the cardiac tissue at a cellular level leading to arrhythmias. An electric field applied to the tissue induces polarization, which is influenced by small structural features such as cell membranes, fluid content, collagen/fibrosis, and myocyte anisotropy. Such dielectric changes after conduction velocity and heterogeneity promote reentry circuits and trigger activity, which form the bases for arrhythmogenesis, guiding diagnosis and appropriate treatment.

The presence of peri-procedural edema causes an increase in the amount of interstitial water and its conductivity; hence, a change in myocardial dielectric properties. This can temporarily mask the evidence of partial tissue injury and cause a conduction block which is reversible. DURABLE-I is a single-center study not randomized which is described in a conference abstract. Through retrospective lesion pair analysis, the algorithm of dielectric lesion assessment showed an accuracy rate of 76.5 percent. They have found that correction of the identified algorithmic gaps before the occurrence of edema can lower the chance of reconnection from 95% to 25% [24].

### 3.2. Intrinsic Dielectric Properties and Catheter-Based Impedance Measurements: Distinctions and Measurement Technologies

It is imperative to make a crucial difference between myocardial dielectric properties and local impedance measured by catheters. Myocardial dielectric properties such as myocardial conductivity and permittivity are frequency-dependent properties of the tissue which are measured using experimental conditions involving the precise geometry of electrodes and tissue preparation. Local impedance (LI), on the other hand, is an apparent electrical property measured by specific electrode configurations involving the composition of the tissue, catheter-to-tissue interface, geometry of the electrode, conductivity of blood pool, thickness of wall, catheter orientation, sensing frequency, temperature, and movement. LI cannot be used to infer intrinsic myocardial conductivity, permittivity or resistivity since they represent entirely different methods of measurement. LI can thus be said to involve a system-dependent measurement of the clinical significance that involves information about the composition of the underlying tissue and lesion formation.

Catheter-based impedance techniques give local measurements of electrical parameters during electrophysiological procedures; however, these cannot be confused with experimental direct measurements of the dielectric properties of tissues. In the case of local impedance measurements, small electric fields are applied through an established arrangement of electrodes, with the measurement being the local impedance. The measurements can be made simultaneously with electrograms, voltage, time of activation, and other procedure parameters [21,23].

In addition, multi-electrode mapping allows for high-quality spatial resolution and creates three-dimensional maps of impedance distribution that consider tissue heterogeneity in regions where mapping was carried out [21]. The combination of impedance and voltage measurements might contribute to the better identification of viable myocardium, fibrotic tissue, and areas of conduction disturbance. Finally, epicardial impedance measurement in surgical or percutaneous access can be used as a method to assess myocardial surface in cases of predominantly epicardial substrate [21].

Clinically, to identify such changes, wide-band dielectric imaging (DI), i.e., the KODEX-EPD system, implements a unique dielectric property of blood and myocardial tissue to generate high-resolution 3D reconstructions without any direct contact between the catheter and tissues. This contact-independent approach may facilitate the identification of tissue-related dielectric differences while reducing reliance on ionizing radiation [63].

Techniques based on impedance have enabled researchers to expand the application of dielectrics beyond the confines of the electrophysiology laboratory. The use of thoracic bioimpedance in monitoring hemodynamic parameters has proven the possibility of obtaining continuous measurements of impedance in real time at the patient’s bedside, even though the resolution of this technique is relatively low [22]. Some other techniques, like electrical impedance tomography (EIT), involve using electrode arrays on the body surface to construct images of cross-sectional impedance distribution inside the thorax. Even though the resolution and signal separation capabilities of EIT still pose some limitations, the concept itself is the key to obtaining non-invasive images of local dielectric variations [22].

Wearable and patch-based impedance monitoring devices can be considered promising instruments that may allow continuous monitoring outside of the laboratory setting. Such devices would provide information regarding changing parameters related to thoracic impedance caused by developing heart disease [22].

While methods such as thoracic bioimpedance, impedance measurement using wearable sensors, and impedance tomography allow non-invasive longitudinal monitoring, these approaches largely provide information about global changes in the fluid content in the thorax, respiration, or hemodynamics. The current resolution of the above-mentioned methodologies is insufficient for assessing the focal properties of myocardial substrate.

### 3.3. Tissue Determinants of Dielectric Signatures

#### 3.3.1. Remodeling and Structural Changes: Fibrosis, Scar, and Border Zone Heterogeneity

Changes in fibrotic and scar tissue affect electrical characteristics measured depending on frequency range, sample preparation, and methods of measuring the electrical parameters. Fibrosis and scar replace living muscle tissue with collagen, damage cellular membranes, and alter extracellular space volume, the anisotropic nature of tissue, and water content. As a result, the existing literature does not show any universal changes in resistivity or impedance at various frequencies and conditions. For instance, in the chronic-infarction model, dense scar was found to exhibit lower resistivity compared to healthy tissue and lose its dependence on frequency, which is characteristic for the latter, while in other experimental setups lower conductivity due to collagen deposition and impaired cellular connections were described [62].

The development of myocardial scarring is a step further from fibrosis in terms of structural myocardium remodeling. It consists of dense nonviable collagenous tissue with a unique electrical profile [22]. The borderline region between the scarred myocardium and healthy tissue contains a heterogeneous combination of viable muscle cells, fibrotic tissue, and inflammatory elements. Consequently, intermediate dielectric characteristics are formed [62]. This area is crucial in electrophysiology since it often harbors regions of slow conduction, which can cause reentrant arrhythmias [62].

#### 3.3.2. Electrophysiological Remodeling: Impact on Dielectrics at Membrane Level

Altered ion channel functioning can influence the tissue’s dielectric characteristics. Ion channel malfunction influences membrane capacitance and conductance, which in turn impacts frequency-dependent impedance characteristics. Those effects become apparent only at high frequencies, when the cellular membrane characteristics become dominant for the measured parameters [22].

Functional issues like conduction system disease and repolarization heterogeneities might alter the tissue’s dielectric signature without the visible presence of structural changes. Progression of fibrosis within specialized myocardial structures, such as sinus nodes and the His–Purkinje system, might cause local changes in impedance characteristics prior to conduction problems developing [22,62].

#### 3.3.3. Inflammatory Processes and Acute Tissue Conditions

Inflammatory changes induce a dynamic and reversible change in tissue dielectric properties. Increased tissue water and cellular infiltrates cause increased conductivity and different permittivities [12]. Acute inflammatory changes evolve over time but might partially reverse, differentiating those from the static and irreversible changes related to chronic fibrotic remodeling.

One of the clinical examples of acute tissue changes affecting impedance properties is peri-procedural edema following catheter ablation. Thermal injury causes inflammation and thus raises tissue water content, causing changes in tissue impedance [23]. However, while those are the most pronounced right after the procedure, the post-procedural remodeling eventually establishes stable dielectric characteristics.

#### 3.3.4. Ischemia, Necrosis, and Dynamics of Tissue Changes

Myocardial ischemia leads to a specific pattern of changes in dielectric properties that differs from scarred heart tissue. Acute ischemia is characterized by cellular swelling and increase in intracellular water, which causes higher conductivity and permittivity [12], whereas the process of chronic infarct formation results in an increase in collagen deposition, decrease in cellularity, lack of complete cellular membrane, and frequency-dependent changes in resistance and capacitance [12,59,60,61,62,64]. Direction and extent of resistivity change depends on the experimental model, sensing frequency, and experimental conditions [12,59,60,61,62,64].

Temperature-dependent changes in tissue anatomy and physiology impact impedance characteristics during ablation procedures. Rise in tissue temperature causes proteins to denature, which increases tissue resistivity. However, those characteristics cannot predict post-procedural changes in lesion impedance, since post-procedural remodeling continues to evolve, see Table 2.

### 3.4. Technical Determinants and Sources of Measurement Variability

The proper interpretation of the results obtained from dielectric studies depends on various parameters. First of all, blood pool conductivity may serve as a shunting factor, especially at lower frequencies, and may be affected by hemodynamics [12]. Cardiac and respiration-induced movement causes impedance signal variability, for which some signal-processing measures should be taken [12,22].

The nature of catheter–tissue interaction plays an important role in the assessment of the measurement’s validity, as variations in catheter contact pressure do not depend on tissue-specific parameters [21]. Furthermore, temperature-related fluctuations during the course of ablation cause significant changes in tissue dielectrics, as they reflect the dynamic process of protein denaturation, cell destruction, and dehydration [23].

## 4. Clinical Evidence and Arrhythmia-Specific Clinical Interpretation

Cardiac tissue exhibits distinct dielectric properties across the different arrhythmias substrates which is summarized in Figure 2A,B. This figure shows how dielectric sensing helps characterize myocardial tissue at the cellular level. It compares dielectric signatures across most common arrhythmia types (AF, atrial flutter/SVT, VT, VF, bradyarrhythmia), each linked to distinct substrate changes like fibrosis, scarring, or edema. Figure 2 depicts the relationships between tissue pathology, measured dielectric properties in experiments, catheter-based impedance measurement and the analysis of these data for possible applications. Figure 2 differentiates the clinical applications of impedance-based methods from hypothetical applications based on AI technology. Classification of tissues, predictions of outcomes and guidance during procedures using either dielectric or LI as input data are still hypothetical or in the preclinical stage.

There is significant variation between the existing studies in terms of laboratory settings, sensing frequency, catheter systems, tissue media, and validation measures. Table 3 illustrates this variation in a comparison of selected studies.

Table 3 outlines the general guidelines for impedance-based tissue characterization and lesion monitoring along with the threshold values that have been identified in the current study. However, these values are conditional on aspects such as catheter type, electrode arrangement, body region, tissue, contact and procedural protocols, among others; thus, they cannot be considered universal clinical threshold values.

### 4.1. Atrial Fibrillation and Atrial Ablation

Atrial fibrillation is the most common sustained cardiac arrhythmia and a leading cause of stroke, heart failure, and cardiovascular hospitalization. The pathophysiological substrate centers on atrial cardiomyopathy, in which progressive fibrosis creates a milieu for abnormal electrical propagation [69,70]. Fibrosis disrupts intercellular coupling, promotes unidirectional conduction block, and enables localized micro-reentry—particularly at the boundaries between fibrotic and non-fibrotic tissue [70]. Reentrant activities cluster around zones of atrial fibrosis, with computational models confirming spatial predilection of drivers to fibrotic borders [70]. Intramural fibrosis, rather than surface fibrosis alone, serves as the structural “fingerprint” of true AF drivers [71]. The transition from paroxysmal to persistent AF is accompanied by increasing fibrotic burden, greater scar transmurality, and more complex reentrant substrates [69,70].

In therapy, dielectric sensing provides real-time guidance using tools like the Tissue Response Viewer. This device evaluates the transmural property of the lesion by observing immediate biophysical changes during ablation. The hypothesis-generating DURABLE-I findings are summarized above and in Table 3 [24]. Prognostically, the WAVE MAP AF study found that an LVA extension greater than 28% of the left atrium in sinus rhythm strongly predicts recurrence, with a hazard ratio of 4.82. Current research focuses on randomized trials like IDEAL AF, which targets LVZs of 3.0 cm^2^ or more, and SUPPRESS AF, indicating that while overall results for LVA ablation are mixed, patients with extensive remodeling or a left-atrial diameter greater than 44 mm see significant benefits [72]. Other new designs, such as IMPRoVED-AF and INVENTION, are testing AI-guided driver mapping and high-density fractionation mapping to make AF therapy more personalized.

#### 4.1.1. Dielectric Signatures in Atrial Fibrillation

Local impedance (LI) mapping using commercially available technology (DirectSense, Boston Scientific) has demonstrated the ability to distinguish pathological from physiological atrial substrate. In full-chamber left-atrial impedance maps, pulmonary vein tissue shows significantly higher impedance elevations above blood pool (median 29–46 Ω) compared to remaining atrial walls (median 16–20 Ω), reflecting the thicker, more muscular PV sleeves [25,73]. Prior ablation lesions are distinguished from their surroundings by a significant drop in local impedance, while corresponding regions in control patients without prior ablation show no such difference [25].

The relationship between LI and atrial fibrosis is complex. When tissue fibrosis is classified by LI itself (rather than bipolar voltage), a significant interaction effect on the contact force–LI relationship emerges: in fibrotic tissue (classified as “electrically inert”), increasing contact force produces minimal changes in LI, whereas in healthier tissue (“electrically active”), LI continues to rise with increasing force. This suggests that LI may reflect tensile and compositional changes secondary to fibrosis that affect catheter–tissue coupling, potentially representing a better reflection of tissue fibrosis than bipolar voltage alone [67].

At the biophysical level, fibrotic myocardium shows lower mean impedance values and more depressed systolic–diastolic dynamic impedance changes compared to normal myocardium. In a chronic-infarction model, mean phasic resistivity decreased progressively from normal to heterogeneous and dense scar regions (285 ± 10, 225 ± 25, and 162 ± 6 Ω·cm at 41 kHz, respectively; *p* < 0.001), with resistivity and phase angle correlating significantly with the degree of local fibrosis (r = 0.86 at 1 kHz; r = 0.84 at 41 kHz) [38]. Dense scar tissue exhibits lower resistivity than normal myocardium (110 ± 30 vs. 235 ± 60 Ω·cm at 1 kHz) and, unlike normal myocardium, shows no capacitative response (no change in resistivity or phase angle with increasing frequency), reflecting the loss of intact cell membranes [74].

Dielectric imaging systems differentiate materials based on conductivity and permittivity at multiple frequencies, establishing dielectric dispersion patterns. In cardiomyocytes, intracellular fluid is an important conductor while the sarcoplasmic reticulum contributes to permittivity; fibrotic replacement disrupts both components [20].

#### 4.1.2. Diagnostic Utility

Substrate mapping: LI mapping can complement voltage mapping for identifying fibrotic substrate and low-voltage zones. The combination of electrogram-based and impedance-based substrate mapping has the potential to improve patient outcomes, as these modalities provide complementary information. LI may be particularly valuable in regions where voltage mapping provides ambiguous results, such as areas with adequate voltage but significant fibrotic remodeling [25].

Gap detection during PVI: At three-month follow-up after PVI, LI measurements can distinguish mature ablated tissue (~107–108 Ω), reconnected segments (~116 Ω), and healthy myocardium (~125 Ω), validating the use of starting LI to identify gaps in ablation lines during repeat procedures [26].

Prediction of durable conduction block: An LI drop ≥13.4 Ω during PVI predicted gap-free lesion formation with 78% sensitivity and 75% specificity. A larger LI drop at the index procedure correlates with a higher probability of achieving durable conduction block at three months [24,27].

#### 4.1.3. Therapeutic Guidance

LI monitoring during RF ablation provides real-time feedback on lesion formation that outperforms generator impedance. Values of LI and the respective thresholds for ablation depend on the catheter system, the sensor array used, the region of the body, the tissue substrate, and the procedure followed in each study. Therefore, these can only be viewed as study-specific relationships. Key thresholds and principles are as follows:

Lesion adequacy: LI drop correlates with lesion depth both in vitro (R = 0.84) and in vivo (R = 0.79). An LI drop > 20 Ω indicates sufficient lesion creation, while drops >65 Ω warn of excessive heating. In preclinical studies, using an LI drop target of 15–30 Ω achieved 100% durable conduction block (16/16 lesion sets) [28,29].

Regional thresholds: Optimal LI drop cut-points vary by atrial wall location—21.8 Ω on the anterior wall (AUC 0.80) and 18.3 Ω on the posterior wall (AUC 0.77). These regional differences likely reflect variations in wall thickness [30].

Temporal dynamics: Early rapid LI drop is critical. An LI drop < 9 Ω at 4 s predicted unsuccessful applications (OR 3.82; *p* < 0.001). In successful applications, LI continues to decrease significantly beyond 2 s (baseline → 2 s → 10 s: 161 → 150 → 141 Ω), whereas unsuccessful applications show no further change after an initial modest drop [31].

Combined parameters: Combined contact force and baseline LI predicted successful RF applications (AUC 0.75) better than either parameter alone (baseline LI AUC 0.72; mean CF AUC 0.60). Baseline LI showed better correlation with LI drop (R = 0.35) than mean contact force (R = 0.17) [32].

Power optimization: Higher-power RF achieves greater LI drops, shorter ablation times, and more transmural lesion formation. High-power settings reach minimum LI approximately 4 s faster than low-power settings, with the temporal advantage widening as the target LI drop increases [33].

Safety monitoring: Steam pops are associated with higher baseline LI (median 148 Ω) and LI drop (median 59 Ω), with the smallest LI drop associated with a steam pop being 37 Ω. A rapid LI drop of 45 Ω was associated with cardiac tamponade in one reported case [34,35].

The CHARISMA pilot study demonstrated the feasibility of LI-guided PVI, representing the first prospective evaluation of this approach [63].

Overall, studies on atrial ablation demonstrate that local impedance provides tissue-specific data which is superior compared to the data from the generator impedance. Baseline local impedance, changes in local impedance, and dynamics of local impedance are related to lesion creation and conduction gap detection [24,26,27,28,29,30,31,32,33,34,35,36]. However, thresholds differ. Changes in local impedance are estimated from 13.4 Ω in the prediction of gap-free lesion creation to different regional thresholds such as 18.3 Ω in the posterior wall and 21.8 Ω in the anterior wall; in experiments, values are 15–30 Ω or above 20 Ω. Such discrepancies may be attributed to differences in baseline impedance, wall thickness, contact force, power delivery technique, anatomical site, and methodology. In most of the studies, acute endpoints or results of repeated interventions are analyzed, while data supporting effectiveness of local impedance-guided ablation in arrhythmias management are relatively scarce. Therefore, local impedance should be viewed as a supplementary procedural parameter.

### 4.2. Atrial Flutter and Other Supraventricular Tachycardia

Typical atrial flutter is a macroreentrant circuit utilizing the cavotricuspid isthmus (CTI), while atypical flutter circuits involve scar-related isthmuses in either atrium [75,76]. Catheter ablation of the CTI with the achievement of bidirectional conduction block is the definitive treatment for typical flutter, with high success rates [76]. However, confirming complete bidirectional block remains challenging as residual slow conduction may mimic complete block if recording and pacing sites are not positioned close to the ablation line [76]. Approximately 14% of patients have fractionated or indeterminate electrograms near the CTI ablation line, making standard pacing maneuver measurements impossible [77].

For AVNRT, the reentrant circuit involves functionally distinct “fast” and “slow” pathways. The slow pathway is located along the inferior Triangle of Koch and can be visualized as a low-voltage bridge (LVB) on voltage mapping [78,79]. Variant pathways include rightward and leftward inferior extensions, with the left inferior extension being less common but clinically important [80]. Accessory pathways in AVRT are extranodal connections across the AV groove that can conduct anterogradely, retrogradely, or both [79].

The direct evidence for dielectric tissue mapping in atrial flutter, atrioventricular nodal reentrant tachycardia, and atrioventricular reentrant tachycardia is scarce. Local impedance can provide valuable information concerning catheter–tissue contact and lesion formation during ablation of the cavotricuspid isthmus; yet, the suggested clinical utility for locating scars in isthmuses, slow pathways, and insertion sites of accessory pathway is mostly based on atrial fibrillation and ventricular scar research.

#### Dielectric Signatures in Atrial Flutter and Other Supraventricular Tachycardias

Direct evidence for dielectric mapping in flutter and SVT is limited, but several principles can be extrapolated:

CTI tissue characterization: Since LI mapping distinguishes scar from healthy atrial tissue, it can theoretically identify the tissue composition of the CTI and scar-related isthmuses in atypical flutter. Regions of fibrosis within the isthmus would show lower LI values, while viable tissue gaps would show higher LI [25].

AVNRT slow pathway: The LVB identified on voltage mapping (0.3–1 mV range) represents a region of distinct tissue composition within the Triangle of Koch. LI mapping could provide complementary tissue characterization of this region, potentially distinguishing the slow pathway substrate from surrounding atrial tissue based on differences in cellular composition and extracellular matrix [78].

Accessory pathways: LI reflects tissue composition independent of wave front direction, suggesting potential utility in localizing pathway insertion sites where myocardial tissue bridges the AV groove. The dielectric contrast between pathway tissue and surrounding fibrous annulus could theoretically be detected [37].

The possible uses include the recognition of remaining conducting pathways inside linear scars and the assessment of scar-dependent atrial isthmuses. But there is no direct proof for the clinical usefulness of the impedance localization of slow pathways or accessory pathways in the area of the atrioventricular node [25,37,78,81,82].

### 4.3. Premature Ventricular Complexes and Idiopathic Ventricular Tachycardia

Idiopathic PVCs and VT arise from focal mechanisms in structurally normal hearts, most commonly from the right and left ventricular outflow tracts, aortic cusps, coronary venous system, papillary muscles, and fascicles [83,84]. Catheter ablation guided by activation mapping and pace-mapping is the standard technique, with success rates of approximately 85% when PVCs are present during the procedure [84]. However, success drops to ~56% when PVC burden is low during ablation, and deep intra-myocardial foci remain challenging to reach [84]. Interestingly, low-voltage areas have been identified in the RVOT of patients with frequent idiopathic PVCs, and discrete diastolic potentials at successful ablation sites may represent a form of triggered activity [84].

According to the present literature on idiopathic premature ventricular complexes and ventricular tachycardia, impedance measurement is mainly used as a marker of catheter–tissue interface and lesion creation but not as a validated tool for the detection of arrhythmogenic tissues. Although noncontact mapping and dielectric imaging can be helpful in the reconstruction of anatomy and minimization of fluoroscopy usage, it does not allow for the creation of unique dielectric signature of arrhythmogenic focus.

#### 4.3.1. Dielectric Signatures in Premature Ventricular Complexes (PVCs) and Idiopathic VT

In structurally normal hearts, the dielectric contrast between focal arrhythmogenic sites and surrounding normal myocardium is expected to be subtle, as there is no significant fibrosis or scar. The primary dielectric signatures relevant to PVC ablation are as follows:

Tissue-blood pool distinction: LI provides clear distinction between blood pool (~67.6–122 Ω) and myocardial contact (~119.7–220 Ω), with the magnitude depending on catheter orientation (perpendicular contact: 220 ± 29 Ω; parallel contact: 207 ± 31 Ω). This contrast is substantially larger than that provided by generator impedance (blood pool ~100.5 Ω vs. myocardium ~123.8 Ω) [28,34,35].

Contact quality assessment: Baseline LI correlates with tissue contact and is unaffected by catheter orientation in vitro. The first clinical use of LI-incorporating catheters demonstrated strong exponential correlation between LI and electrogram amplitude in both LV and LA [35,85].

Subtle substrate markers: The low-voltage areas and discrete diastolic potentials identified at PVC origin sites may have corresponding LI signatures, though this has not been directly studied [84].

#### 4.3.2. Diagnostic Utility

Catheter–tissue contact confirmation: LI provides superior contact assessment compared to generator impedance, which is particularly valuable in the outflow tracts where catheter stability can be challenging due to cardiac motion and complex anatomy.

Rapid localization: In the absence of spontaneous PVCs, LI-based tissue characterization could potentially identify subtle substrate abnormalities at the site of origin, complementing pace-mapping when activation mapping is not possible.

Fluoroscopy reduction: Reliable LI-based contact confirmation could reduce dependence on fluoroscopy for catheter positioning, contributing to radiation dose reduction.

#### 4.3.3. Therapeutic Guidance

Lesion formation monitoring: Standard practice targets an impedance drop of ≥10–15 Ω or ~10% from baseline during outflow tract ablation. LI monitoring provides more sensitive and specific feedback than generator impedance for this purpose [86,87].

Ablation adequacy: LI drop correlates with lesion depth more strongly than generator impedance drop (R^2^ = 0.82 vs. 0.58 for depth), enabling more precise titration of energy delivery in thin-walled structures like the RVOT where the risk of perforation must be balanced against the need for transmural lesions [35].

Safety: LI monitoring can warn of excessive heating before steam pops occur, which is particularly important in the outflow tracts given proximity to coronary arteries and the pericardial space.

### 4.4. Scar-Related Ventricular Tachycardia and Post-Infarction Substrate

Scar-related VT is the most common sustained ventricular arrhythmia in patients with structural heart disease. The mechanism is macro-reentry through surviving myocardial fiber bundles within areas of fibrous scar, with initial events commonly occurring years after the acute MI [88]. The substrate is characterized by regions of slow conduction, unidirectional conduction block, and areas of fixed or functional conduction block that define the reentry path [88]. For most post-MI VTs, the substrate is subendocardial, though circuits can also involve the midmyocardium and epicardium [88,89].

The chronic scar pattern extends from the sub-endocardium with variable transmurality. Along the border, viable myocardium interspersed with fibrous tissue provides the substrate for slow conduction [88]. Channels of surviving myocardium within the scar—identified by isolated potentials, late potentials, and very late potentials—represent critical components of the reentrant circuit [90]. Both the extent of the very-low-voltage zone and the presence of numerous channels within this zone are critical to the development of ventricular arrhythmias [90].

A critical and underappreciated component of the VT substrate is lipomatous metaplasia (LM)—the replacement of fibrosis by adipose tissue within the infarcted myocardium. LM enables VT through multiple mechanisms: it reduces current loss within protected corridors (maintaining stable current amplitude along the corridor length), facilitates slow conduction in critical VT corridors (median CV 8.8 vs. 39.2 cm/s in non-critical corridors; *p* < 0.001), and increases repolarization dispersion [91,92,93]. Of 94 corridors confirmed to participate in VT circuitry, 93 traversed through or near LM [92]. LM incidence, like monomorphic VT, occurs years after infarction, suggesting it may be an important determinant of VT circuit evolution [91,94].

Current approaches in catheter ablation for post-MI VT have moved from voltage-based approaches toward the identification of regional dielectric heterogeneity and slow-conduction corridors as potential targets. Voltage-based approaches have difficulty in differentiating between inert scar and viable arrhythmogenic tissue because of the dependency of the approach on the direction of the wave front propagation and the inability of the approach to detect small electrodes. In contrast, dielectric-based approaches, like EnSite X with Omnipolar Technology, improve the resolution of the infarct gray zone (0.5 to 1.5 mV) [95,96]. Recent multicenter study results have shown that VT ablation in the zone and Evoked Delayed Potentials (EDPs) unmasked by double extrastimuli improve 12-month VT-free survival (up to 88%) and increase acute non-inducibility compared to standard approaches for scar homogenization [97,98].

#### 4.4.1. Dielectric Signatures in Scar-Related VT and Post-MI Substrate

Local impedance mapping of VT substrate: In a landmark post-MI swine model study, Aranyó et al. demonstrated that VT isthmuses consistently localize to regions of intermediate LI, regardless of voltage amplitude. Specific LI distribution patterns were associated with VT inducibility: corridors of intermediate LI between regions of low LI, LI gradients, and high-LI heterogeneity all predicted critical VT sites. Critically, in cases where voltage mapping showed a large, seemingly homogeneous area of dense scar with no identifiable voltage channels, LI mapping revealed significant tissue heterogeneity including corridors of intermediate impedance that colocalized precisely with the VT isthmus [37].

Histological correlates: In a follow-up study, the tissue characteristics underlying different LI subtypes were elucidated. Low-LI zones correspond to dense transmural scar with pronounced wall thinning, while intermediate-LI tissue shows predominantly subendocardial scarring with more heterogeneous composition. Intermediate-LI zones display less collagen and greater vascular density compared to low-LI zones. Importantly, 75.6% of CMR-defined border-zone corridors colocalized with intermediate-LI tissue [38].

Clinical VT ablation data: In clinical VT ablation, baseline LI was significantly lower in scarred myocardium (87 Ω) compared to healthy myocardium (97.5 Ω), while generator impedance showed no difference between tissue types. The LI drop during ablation was higher for VT-terminating applications (18 Ω vs. 13 Ω; *p* = 0.03), and the LI drop rate was also higher (0.63 vs. 0.32 Ω/s; *p* = 0.008) [39].

Biophysical basis: Myocardial resistivity and phase angle correlate significantly with the degree of local fibrosis (resistivity: r = 0.86 at 1 kHz; phase angle: r = 0.84 at 41 kHz) [59]. Normal myocardium shows phasic systolic–diastolic impedance changes (amplitude 18 ± 2 Ω·cm at 41 kHz) that are markedly depressed in dense scar (10 ± 1 Ω·cm) [59]. The infarct scar is a low-resistance, non-capacitative medium (110 ± 30 vs. 235 ± 60 Ω·cm for normal myocardium at 1 kHz), reflecting the loss of intact cell membranes and replacement by connective tissue [74].

Lipomatous metaplasia and impedance: LM exhibits higher electrical impedance than fibrotic scar but lower than normal myocardium. This higher impedance reduces current dissipation into surrounding tissue, effectively protecting the electrical impulse as it propagates through narrow surviving myocardial channels and enabling sustained reentrant VT circuits. Corridors adjacent to LM maintain relatively stable current amplitude along their length (low standard deviation), while corridors traversing scar alone exhibit substantial current loss [74]. The distinct impedance of LM from scar provides a potential target for dielectric-based tissue characterization that could identify this arrhythmogenic substrate.

Computational modeling: When the infarcted zone is modeled as a mixture of normal tissue, fibrosis, and fat, the zone is expected to be less conductive than normal tissue, since both fibrosis and fat are less conductive than normal tissue. This contrasts with the globally measured conductivity of infarcted tissue (which appears more conductive than normal), highlighting the importance of understanding the heterogeneous composition of scar at the tissue level [99].

#### 4.4.2. Diagnostic Utility

Substrate characterization beyond voltage: LI mapping provides a complementary dimension to voltage mapping for identifying the arrhythmogenic substrate. In cases where voltage mapping shows homogeneous dense scar, LI can reveal tissue heterogeneity and identify corridors that support reentry [37].

Border-zone delineation: The ability to distinguish dense scar (low LI), border zone (intermediate LI), and normal myocardium (high LI) provides a three-tier tissue classification that refines the binary scar/no-scar distinction of voltage mapping [37,38].

Identification of LM-associated substrate: Given the distinct impedance properties of LM compared to fibrosis, dielectric mapping could potentially identify LM-rich regions that harbor critical VT corridors, complementing CT-based LM detection [91,92].

Prediction of VT inducibility: Specific LI distribution patterns (corridors, gradients, high heterogeneity) are associated with VT inducibility, suggesting that LI mapping could contribute to risk stratification [37].

#### 4.4.3. Therapeutic Guidance

Ablation target identification: Intermediate-LI regions that form corridors between low-LI zones represent potential ablation targets, even when voltage mapping does not identify clear channels [37].

Ablation endpoint assessment: LI drop during ablation is higher for VT-terminating applications, suggesting that LI monitoring can help identify effective lesions in real time. The LI drop rate provides additional temporal information about lesion efficacy [39].

Dense scar vs. border-zone differentiation: Understanding whether a region represents dense transmural scar (low LI, pronounced wall thinning) or heterogeneous border zone (intermediate LI, subendocardial scarring) can guide decisions about where to place ablation lesions and where ablation is unlikely to be effective [38].

Integration with existing strategies: LI mapping can be integrated with established substrate ablation approaches such as targeting deceleration zones, late potentials, and scar channels, providing an additional biophysical parameter to refine target selection [90,100,101].

There is a strong scientific consensus within VT studies regarding the role of local impedance (LI) in revealing tissue heterogeneities that cannot be easily observed using standard voltage maps. In experimental research, the presence of an intermediate-impedance pathway has been related to VT isthmuses, while low- and intermediate-impedance areas have been correlated to dense transmural scar and heterogeneous subendocardial scar, respectively. Clinical research has shown a correlation between lower LI in scar tissue and lower LI decreases in sites of VT termination during ablation procedures. Yet, the size of the published studies has been rather small, different criteria for classifying impedance subtypes have been used, and most of the studies were performed in the context of ischemic scars.

### 4.5. Ventricular Fibrillation and Polymorphic Ventricular Tachycardia

Ventricular fibrillation (VF) and polymorphic ventricular tachycardia (VT) arise through complex mechanisms involving acute triggering events, ischemia, cardiomyopathy, channelopathies, and the dispersion of myocardial refractoriness. Unlike monomorphic VT related to myocardial scarring, VF usually does not involve an identifiable focal reentry circuit characterized by the presence of localized dielectric features [89,102].

Empirical data on the dielectric myocardial mapping in VF or polymorphic VT is sparse. Impedance studies in these conditions have mainly been done using intrathoracic impedance from implanted devices. For a population of more than 2000 subjects with Implantable Cardioverter Defibrillators (ICDs), the reduction in intrathoracic impedance was associated with the detection of VT or VF; however, impedance reduction was seen without any arrhythmia, and only a few cases had such a reduction prior to the arrhythmia [103,104]. Intrathoracic impedance is a marker of fluid overload and hemodynamics, but not a focus in the myocardium, and therefore it cannot serve for arrhythmia risk prediction [105,106,107].

The transthoracic impedance determines the effectiveness of energy delivery during defibrillation, and identifiable ventricular premature beats can be mapped and ablated through standard activation mapping. However, these techniques are different from dielectric tissue characterization. Currently performed studies of dielectric properties in the assessment of risk for VF, optimization of defibrillation energy, or triggering mechanisms should be considered indirect or experimental.

### 4.6. Bradyarrhythmias and Conduction System Disease

Conduction disorders such as sinus node dysfunction, atrioventricular block, and His–Purkinje disease can be caused by fibrosis, fatty infiltration, calcification, or the ischemic damage of the specialized conduction system. These pathologies explain the biological grounds for dielectric tissue characterization; however, there are no studies on the dielectric properties of nodal or conduction system tissue.

Impedance is already used in cardiac implantable electronic devices for lead integrity control and physiological sensing. Sudden or gradual changes in impedance of pacing can be used to detect conductor fracture, insulation failure, or changes in the lead–myocardium interface [73]. Closed-loop pacing systems use changes in intracardiac impedance as surrogates of ventricular contractility, and impedance-based hemodynamic signals are being evaluated to optimize AV and interventricular delays in cardiac resynchronization therapy [108,109,110]. In all these cases, device circuit impedance or hemodynamic impedance are measured, but not the dielectric properties of arrhythmogenic myocardial tissue.

Locally measured impedance can theoretically help in conduction system pacing for the identification of viable myocardium, fibrosis, or the lead–tissue interface. However, its capability of improving His bundle or left bundle branch area pacing, lead longevity, and clinical outcomes remains to be prospectively validated. Therefore, this application can be considered hypothetical rather than an existing one.

### 4.7. Inherited Channelopathies

The cases of Long-QT syndrome, catecholaminergic polymorphic ventricular tachycardia, and early repolarization syndrome include functional channelopathy with no consistent macroscopic structural anomalies. Thus, it would be expected that there are no significant dielectric differences between affected and healthy myocardium in current technological conditions.

Brugada syndrome might be considered an exception to the mentioned rule. There are studies on the histological characteristics that included fibrosis, inflammation, decreased connexin-43 expression, and conduction disturbances within the right ventricular outflow tract, specifically the epicardium of this area [111,112]. Such characteristics give a possibility to consider the dielectric mapping of potentially abnormal myocardial areas which form the Brugada syndrome substrate. However, the existing data regarding epicardial ablation is mainly based on the results of electrograms and conduction mapping.

Artificial intelligence applied to the analysis of surface ECGs allows for detecting hidden long-QT syndrome phenotypes and genotype variations [113,114,115]. It is an example of adjacent evidence of AI and should not be considered as the evidence of AI-assisted dielectric mapping. In current conditions, dielectric mapping in inherited channelopathies can be considered only as theoretical research area, with the only possible practical application being structural substrate evaluation in the Brugada syndrome.

### 4.8. Pediatric and Fetal Arrhythmias

Pediatric arrhythmias range from structurally normal hearts with supraventricular tachycardias to scar-related arrhythmias in congenital heart disease. Dielectric interpretation may differ from adult experience as a result of developmental variation in myocardial water content, extracellular matrix structure, cellular architecture, and electrical properties. Repaired congenital heart disease may produce dielectric signatures like that seen in adult scar-related substrates by virtue of surgical scars, patch material, and heterogenous fibrosis [37,38]. Arrhythmogenic substrates in structurally normal pediatric hearts will likely show minimal dielectric contrast. Local impedance sensing may assist catheter contact verification and lesion characterization in pediatric ablation, but dedicated pediatric validation is not available at present.

While EIT has been explored mostly for monitoring lung ventilation and perfusion in neonates and children [116,117,118,119,120,121], cardiac-derived impedance changes have been found but the current spatial resolution is inadequate to characterize focal arrhythmogenic substrates. Even greater challenges are encountered in fetus studies due to intervening maternal tissues. Therefore, pediatric and fetal applications of myocardial dielectric mapping have been inferred from adult ablation and impedance monitoring studies.

## 5. Artificial Intelligence in Electrophysiology: Adjacent Evidence and the Limited Evidence for Direct Dielectric Integration

AI is becoming increasingly useful in cardiac electrophysiology, which includes arrhythmia recognition, electrogram analysis, cardiac image analysis, procedural planning, and the prediction of outcomes [40,41,42]. However, there is a paucity of clinical evidence on AI models designed on the basis of myocardial dielectric characteristics and/or impedance measurement. The available literature can be divided into two different tracks: studies on the evaluation of dielectric/impedance sensing without AI interpretation and AI analysis of ECGs, intracardiac electrograms, electroanatomical mapping, or cardiac imaging without the inclusion of dielectric parameters. AI–dielectric integration should currently be regarded as a hypothesis-generating translational research direction rather than an established clinical strategy.

The future role of AI-based dielectric monitoring has to be assessed in light of current limitations in sensing technology. Local impedance measurements of the myocardium rely on invasive intracardiac catheterization during electrophysiological investigations and are therefore not ready for use in ambulatory substrate monitoring. Non-invasive longitudinal assessment may be performed using thoracic bioimpedance, impedance-sensing wearables, and electrical impedance tomography; nonetheless, the above-mentioned methods provide information about the overall state of the thorax, respiration, and hemodynamics and are not able to provide enough spatial resolution for identifying arrhythmogenic myocardial substrates [22,116,117,118,119,120,121], see Figure 3.

### 5.1. Indirect Evidence: AI Applications Adjacent to Dielectric and Impedance Sensing

The studies summarized in this subsection do not constitute evidence for AI analysis of myocardial dielectric or local impedance measurements; rather, they demonstrate the broader feasibility of AI-based analysis within electrophysiology. AI methods have been tested in various aspects of cardiac electrophysiology that are adjacent to dielectric tissue characterization, including automated ECG interpretation, the analysis of continuous rhythm-monitoring signals, electrogram-based substrate identification, cardiac imaging segmentation, risk stratification, and computational modeling [40,41,42].

The best evidence comes from the TAILORED-AF randomized trial, where patients with persistent or long-standing persistent atrial fibrillation (AF) underwent either pulmonary vein isolation (PVI) or PVI plus ablation of areas with AI-detected spatiotemporal electrogram dispersion. At 12-month follow-up, there was higher freedom from documented AF in patients undergoing AI-guided ablation than in those with PVI alone; nevertheless, procedure and ablation times were longer, and freedom from atrial arrhythmias did not differ between the two groups [43]. Importantly, AI algorithm assessed intracardiac electrograms but not dielectric or local impedance properties of tissues. Hence, the trial proves the feasibility of AI-based substrate analysis but not its validation as an AI-enabled dielectric fingerprinting. In published results, the trial randomized 370 patients and showed 88% versus 70% freedom from AF at one-year follow-up.

Cardiac digital twins and computational modeling provide further evidence. Digital twin models integrate patient-specific anatomy and electrophysiology to simulate mechanisms and the treatment of arrhythmias [44]. In case of scar-dependent ventricular tachycardia (VT), personalized heart models constructed using late gadolinium enhancement cardiac magnetic resonance (LGE-CMR), anatomical, and electrophysiological data were able to identify regions with abnormal electrograms and conduction delays and predict potential ablation targets [45,46]. However, these models were usually created on the basis of imaging, anatomical, and electrophysiological data without any myocardial dielectric data. The integration of impedance-derived tissue information into these models is a future prospect and not a clinically validated technique. In prospective digital twin study, 80.8% of invasively mapped deceleration zones were identified in 18 patients, while evidence on AI-based guidance of ablation is still early feasibility study.

### 5.2. Direct Evidence for AI Using Myocardial Dielectric or Local Impedance Inputs

There is relatively scarce evidence for AI models that are trained specifically on myocardial dielectric or local impedance signals. Majority of clinical local impedance studies have used predefined parameters such as baseline local impedance, absolute impedance drop, impedance-drop rate, and temporal impedance dynamics and did not use any machine-learning techniques [24,26,28,31,35,37,38,39].

The limited direct evidence currently consists predominantly of experimental and ex vivo studies. Impedance measurements at multiple frequencies were shown to have frequency- and temperature-dependent behavior during RF ablation and might help in distinguishing reversible and irreversible thermal changes [47]. Impedance measurement by multichannel impedance configuration has also improved correlation between measured impedance changes and dimensions of lesions compared to conventional generator impedance [48]. These studies proved that impedance signals contain information about lesion formation, but they did not prove the clinical benefit of AI-based impedance signal interpretation.

More recently, Takigawa et al. have developed an extreme gradient-boosting machine-learning model to estimate RF lesion dimensions based on data from 1142 ablations performed in excised ventricular myocardium. Procedural factors such as ablation energy, contact force, catheter orientation, ablation duration, and impedance change were included in the model, which were able to estimate lesion dimensions more accurately than any conventional individual index [49]. This study provides direct evidence of the possibility of the extraction of additional information from the impedance-containing ablation dataset using machine learning, but it was performed in ex vivo setting, did not identify arrhythmogenic tissue, and did not assess any clinical benefits.

The tissue composition affects impedance behavior during ablation. Impedance drop for particular energy delivery may differ between normal myocardium, low-voltage myocardium, and dense scar, suggesting that a single universal impedance threshold may be inappropriate for heterogeneous substrates [1,68]. Future AI models could potentially account for these nonlinear and substrate-specific effects; however, this capability has not been demonstrated in prospectively validated clinical models using dielectric or LI inputs, see Table 4.

### 5.3. Hypothesis-Generating Framework for Future AI–Dielectric Research

As summarized in Table 2, the model below should be viewed as a hypothesis-generating tool and not a clinically validated technique. It has been shown through current research that tissue pathology may affect dielectric and impedance values, but these values are dependent on the frequency of sensing, geometry of measurement, tissue preparation, catheter contact and platform used. Furthermore, there is no known “dielectric fingerprint” or disease specificity which allows for the classification of arrhythmogenic substrates in vivo.

In case reproducibility and validation of dielectric or LI characteristics can be shown independently, machine-learning algorithms may be developed to see whether there is additional information offered by these measures independent of electrograms, voltage mapping, imaging, catheter contact measures and other procedural variables. Possible areas of application would include tissue classification, conduction gap recognition, lesion durably, and substrate assessment. The applications mentioned above should be viewed as investigational at this time [61,64].

Prospective AI-assisted dielectric characterization system may use multistage analytical workflow. Initially, raw impedance or dielectric signals will be corrected for catheter contact, catheter orientation, cardiac and respiratory motion, blood pool conductivity, sensing frequency, tissue temperature, and signal features specific to a particular platform. Then features will be extracted from baseline impedance, frequency-dependent conductivity and permittivity, temporal impedance changes during ablation, spatial impedance gradients, and regional heterogeneity.

The process of identification and correction of artifacts is necessary before the use of AI algorithms in interpretation. The methods for artifact correction are not automatically performed by AI algorithms; rather, they can involve signal processing and machine-learning algorithms which are capable of detecting, marking, correcting, or even ignoring unreliable measurements. There are many potential approaches, such as contact force thresholding, catheter stability thresholding, synchronization with breathing and heartbeat cycles, motion correction, blood pool signal correction, platform-specific correction, temporal filtering, and automatic outlier detection. It is important to consider measurements performed under insufficient contact, catheter displacement, excessive noise, or instable breathing with low confidence or even ignore them [40,41,42].

These dielectric features may be then integrated with bipolar and unipolar voltage, electrogram morphology, activation time, conduction velocity, contact force, ablation power and duration, CT, CMR, electrocardiography, and patient characteristics [40,41,42,122]. Outputs of the model may include the classification of viable myocardium, fibrosis, dense scar, edema, and acute thermal injury; detection of conducting channels in the scar; prediction of lesion durability; and patient-specific risk stratification.

This is a hypothetical framework, and its clinical usefulness depends on the ability of dielectric features to give reproducible and additional information in comparison with established electroanatomic mapping, imaging, electrogram analysis, and procedural parameters. Performance of a multimodal model should be compared not only with histopathology or imaging, but also with clinically meaningful endpoints such as acute conduction recovery, chronic lesion durability, arrhythmia recurrence, procedural efficiency, and complications.

### 5.4. Requirements for Clinical Translation

Several issues have to be solved before AI-assisted dielectric characterization can be implemented in routine electrophysiology practice. First, protocols for signal acquisition, sensing frequencies, definitions of impedance, and reporting standards should be harmonized for different catheters and mapping platforms. Second, model development studies should involve large and diverse populations, predefined procedures for training and validation, independent external validation, and the transparent reporting of missing data, class imbalance, calibration, and model uncertainty [40,41,42].

Construction of a clinically valid model based on AI and dielectrics requires a dataset that is very large and has a good annotation system including raw dielectric signals, acquisition information, electroanatomical mapping, imaging, standardization of procedure, and follow-up data from the procedure. However, such datasets are rare because of dielectric measurements that are unique to platforms, with collection mostly in invasive procedures, and non-standardized storage of data [40,41,42].

Dielectric predictions should be validated against proper reference standards that include histopathology, LGE-CMR, electroanatomic mapping, conduction recovery, chronic lesion formation, and clinical outcomes. Finally, prospective trials should prove the added value of AI-based interpretation in comparison to conventional voltage mapping, electrogram analysis, imaging, ablation indices, and predefined local impedance thresholds.

Other issues include model interpretability, confidence estimates, real-time computational performance, data interoperability, cybersecurity, patient privacy, regulatory oversight, bias evaluation, and post-deployment monitoring for model drift [40,41,42]. Until these requirements are fulfilled, AI-dielectric integration should be investigational and should supplement rather than substitute established electrophysiological endpoints and clinical judgment. Recent EHRA/HRS and HRS statements emphasize reproducibility, interoperability, transparency, external validation, monitoring, recalibration, and responsible clinical implementation.

As far as we know, there have not been any studies done in an external validation in which the principal input of the AI model is the dielectric properties of myocardium or local impedance.

### 5.5. Practical Use: When AI May and May Not Be Applied

Current applications of AI in cardiology have been clinically validated for several areas, such as ECG analysis, rhythm-monitoring analysis, intracardiac electrogram analysis, image segmentation, procedure planning, and specific cases of electrogram-driven ablation of atrial fibrillation. Impedance measurement can act as an additional parameter for lesion formation, pulmonary vein conduction gap identification, and some specific post-myocardial infarction ventricular substrate characterization. Nevertheless, the direct integration of AI with myocardial dielectric measurements is still under investigation. The AI–dielectric models are currently not suitable for clinical use in procedures of lesion localization, ablation targeting, and decision-making. Current applications of AI to atypical atrial flutter, other supraventricular tachycardias, idiopathic ventricular tachycardias, bradyarrhythmias, conduction system diseases, genetic channelopathies, and pediatric arrhythmias rely largely on indirect or conceptual validation. In the case of genetic channelopathies, the use of AI algorithms for ECG analysis is potentially helpful for screening and phenotyping; however, dielectric mapping has not been validated, with possible use only in a research setting for structural alterations associated with Brugada syndrome.

## 6. Limitations

Several limitations should be considered in evaluating this review. First, the paper is a structured review rather than a systematic review or meta-analysis. Despite the fact that several databases and reference lists were analyzed, potential bias in study selection and interpretation cannot be completely ruled out. Significant heterogeneity of studies based on different sensing frequency, electrode arrangement, catheter platform, sample preparation, study participants, and results prevents any quantitative analysis and cross-technology comparison.

Second, the level of quality and clinical applicability of evidence for myocardial dielectric characterization is highly inconsistent. Much of the mechanistic literature is based on ex vivo studies of tissues, animals, computational modeling, or small-scale and single-center trials. While providing valuable information on biological connections between myocardial dielectric properties and fibrosis, scar, edema, ischemia, myocardial temperature, and tissue damage, such studies may not be fully applicable to human electrophysiology. The dielectric signals are also influenced by catheter contact and positioning, blood pool conductivity, myocardial motion, breathing, sensing frequency, and temperature, making the application of proposed thresholds questionable.

Third, the level of direct clinical evidence differs significantly for different arrhythmia types. In particular, most advanced evidence base refers to the use of local impedance measurement for AF ablation and characterization of post-infarction ventricular substrate. On the contrary, applications of dielectrics to atrial flutter, supraventricular tachycardias, idiopathic ventricular arrhythmias, bradyarrhythmias, conduction system disease, inherited ion channel disorders, pediatric arrhythmias, and non-invasive substrate monitoring rely mostly on indirect evidence or physiologic reasoning. Thus, inferences regarding those areas should be considered hypothesis-generating.

Fourth, the evidence for direct integration of artificial intelligence and dielectric signal processing is especially limited. The vast majority of AI studies mentioned in this review involved electrocardiography, intracardiac electrograms, cardiac imaging, wearable sensors, or computational models without taking into account myocardial impedance, conductivity, and permittivity. On the other hand, most of the dielectric studies were based on conventional statistics and predetermined impedance thresholds rather than machine-learning algorithms. While this demonstrates technological compatibility of the two fields, it does not prove their clinical efficacy.

Fifth, most AI algorithms and multimodal frameworks suggested in this review have not yet been validated prospectively in multicenter studies with varied equipment, mapping systems, centers, and patient groups. It is unclear whether they provide additional value beyond voltage mapping, electrograms, imaging, and known procedural metrics. Regulatory approval, interoperability, standardization of data, explainability, and real-time deployment remain to be solved issues.

Finally, publication bias may favor studies demonstrating successful tissue discrimination, good procedural correlation, or new applications of technology. The negative results, failed algorithms, and device-specific problems may be understated. Thus, this review should be considered an overview of existing evidence and research gap rather than proof of efficacy of dielectric fingerprinting or AI–dielectric integration.

## 7. Future Directions

First, there must be evidence for the reproducibility, biological validity, and incremental clinical utility of dielectric and impedance measurements on their own. Efforts will include harmonizing terminology and acquisition protocols for sensing, identifying specific platform or frequency-dependent properties, validating against histopathology, LGE-CMR, electroanatomic mapping, chronic lesion stability, and clinical outcomes. The fact that catheter-based LI is not directly comparable to a tissue dielectric measurement means that cross-platform studies will have to elucidate which aspects of impedance measurements relate to tissue composition and which are related to catheter contact, geometry, blood pool, temperature, etc.

After reproducible dielectric or LI-based metrics are validated, studies need to investigate whether AI models incorporating such measurements offer any incremental value over currently accepted electrograms, voltage maps, imaging modalities, contact force measurements, and impedance-based indices. At this time, it is a research project rather than a clinical application.

## 8. Conclusions

Dielectric characteristics of the myocardium allow an approach to the mechanisms underlying the impact of tissue structure and remodeling on electrophysiological properties. Local impedance via catheter gives clinically available information about the tissue-specific features in the electrophysiology procedures, especially in lesion evaluation and certain atrial and post-infarction ventricular substrates. Nevertheless, local impedance is an operator- and condition-dependent parameter and should not be confused with myocardial intrinsic conductivity, permittivity, or resistivity.

The rationale for the use of AI in combination with dielectric or local impedance measurements is insufficient and mostly based on experimental or ex vivo observations. AI algorithms that have been applied in the clinical electrophysiology practice utilize ECG, electrograms, imaging, or modeling approaches and thus indirectly support further use of AI in dielectric studies. AI-assisted dielectric measurement should thus be regarded at this moment as a hypothesis-generating tool rather than a clinically validated one. The standardization of measurement techniques, validation of biological and external validity of AI algorithms, and multicenter prospective studies are necessary for their implementation into clinical practice.

## Figures and Tables

**Figure 1:**
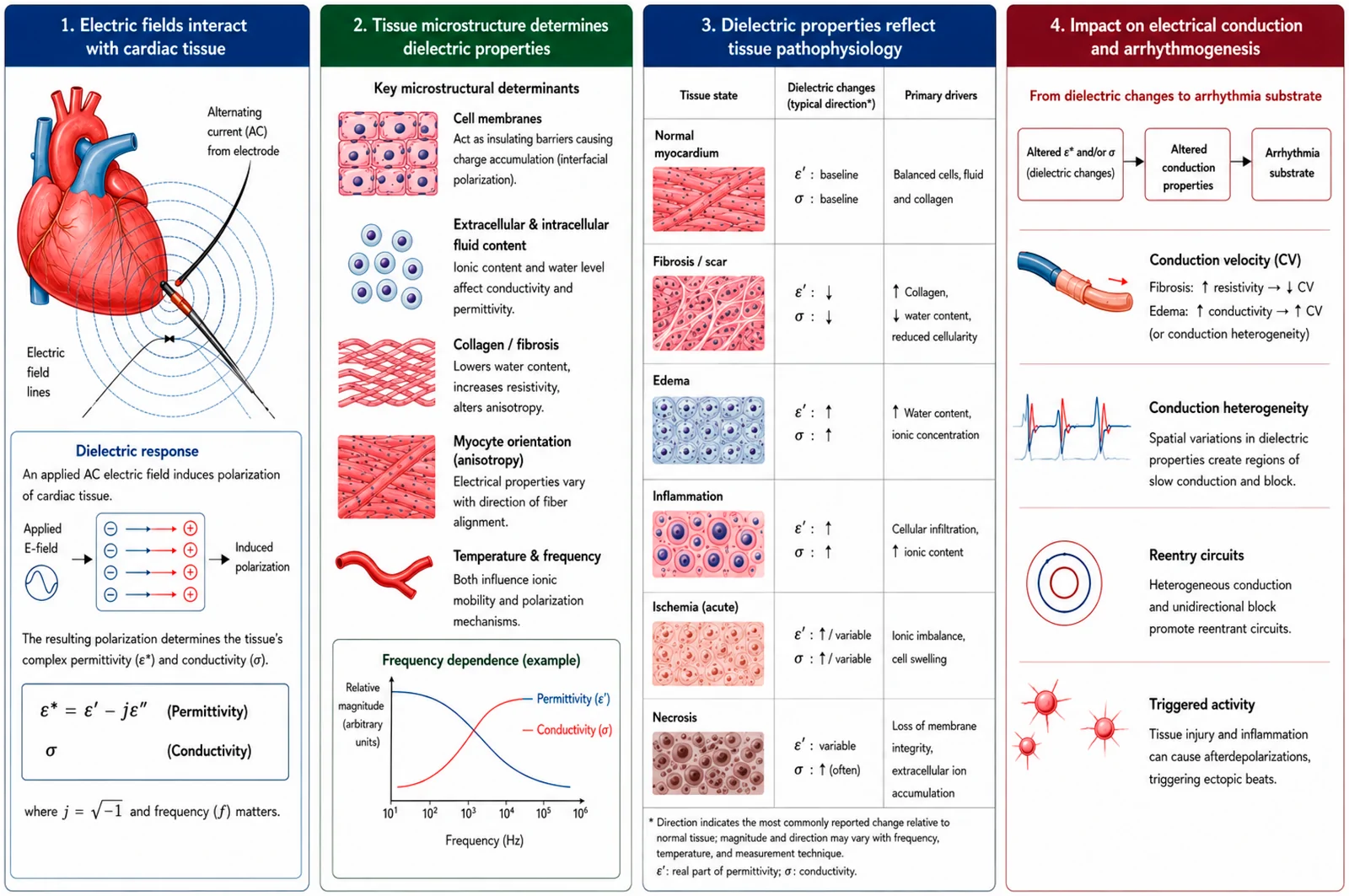
Biophysical determinants of myocardial dielectric properties and their electrophysiological consequences. Author-created schematic synthesized from the biological and dielectric principles reported in references [12,22,50,51,52,53,54,55,56,57,58,59,60,61,62].

**Figure 2:**
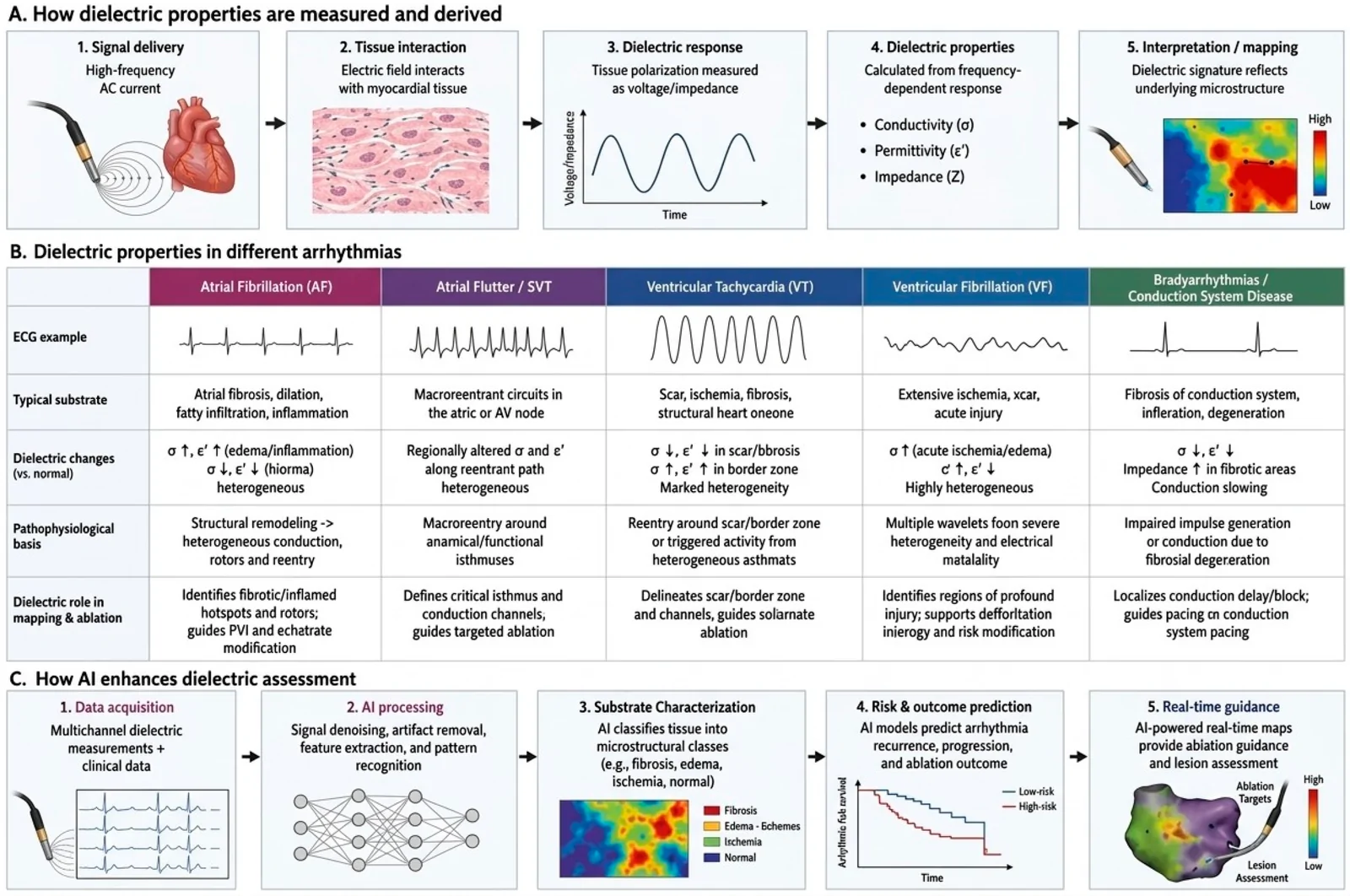
Measurement and interpretation of myocardial dielectric properties and the maturity of arrhythmia-specific applications. Applications are categorized as clinically supported, emerging, or conceptual according to the evidence summarized in references [21,22,23,24,25,26,27,28,29,30,31,32,33,34,35,36,37,38,39,59,60,61,65,66].

**Figure 3:**
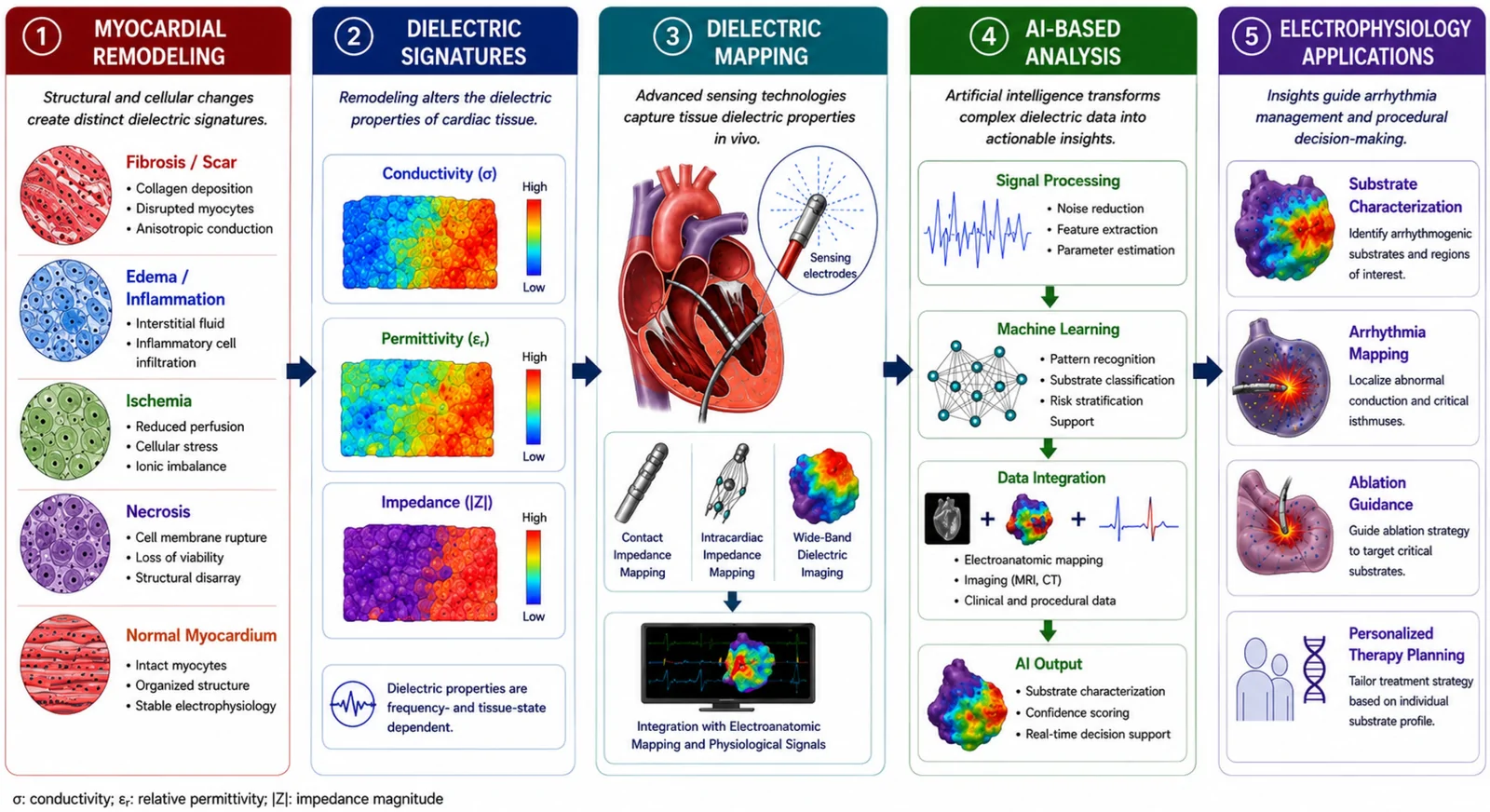
Conceptual framework for AI-assisted dielectric characterization of myocardial tissue. Currently available sensing and analytical technologies are distinguished from proposed AI–dielectric integrations requiring prospective clinical validation. Author-created schematic synthesized from references [40,41,42,43,44,45,46,47,48,49].

**Table 1:** Maturity of evidence for dielectric sensing and AI-assisted applications in cardiac electrophysiology. Authors’ synthesis of the evidence reviewed in references [21,22,23,24,25,26,27,28,29,30,31,32,33,34,35,36,37,38,39,40,41,42,43,44,45,46,47,48,49].

Application	Principal Technology	Evidence Setting	AI Directly Applied to Dielectric Data?	Evidence Category	Current Interpretation
AF lesion assessment	Local impedance monitoring	Human clinical studies	No	Direct clinical evidence	Supported as an adjunct for assessing lesion formation
PVI gap detection	Local impedance and dielectric sensing	Human clinical studies	No	Direct clinical evidence	Promising for identifying residual and reconnected tissue
Post-infarction VT substrate characterization	Local impedance mapping	Human clinical and translational studies	No	Direct clinical evidence	Promising for detecting scar heterogeneity and critical corridors
Multi-frequency lesion assessment	Bioimpedance spectroscopy	Ex vivo and experimental studies	Limited	Preclinical evidence	Demonstrates biological and technical feasibility
AI-guided AF ablation	Electrogram-based AI	Randomized clinical study	No dielectric input	Indirect clinical evidence	Supports AI-assisted EP but not AI–dielectric integration
Imaging-derived cardiac digital twins	CMR and computational modeling	Early human clinical studies	No dielectric input	Indirect clinical evidence	Potential platform for future dielectric integration
Atrial flutter, SVT, and conduction system disease	Proposed LI-based characterization	Mainly extrapolated	No	Conceptual evidence	Requires dedicated clinical validation
AI-based dielectric tissue classification	Multimodal machine learning	Ex vivo or proposed	Potentially	Preclinical/conceptual	Investigational

**Table 2:** Pathophysiological determinants of myocardial electrical properties and their relationship to catheter-based local impedance.

Pathology/ Tissue State	Intrinsic or Experimental Dielectric Findings	Catheter-Based Local Impedance/ Clinical Observations	Electrophysiologic Relevance	Current Interpretation/ Limitations
Dense chronic scar	Chronic infarct scar demonstrates altered frequency-dependent resistivity, conductivity, phase angle, and capacitive behavior. In some experimental models, dense scar has lower resistivity than normal myocardium and shows the loss of the normal frequency-dependent capacitive response [59,60].	Clinical and translational studies have reported lower baseline LI in dense scar than in viable myocardium. Low-LI regions have also been associated with dense transmural scar [37,38,39].	May assist identification of dense scar and differentiation of scar from viable or heterogeneous myocardium during substrate mapping.	Intrinsic resistivity and catheter-measured LI are not quantitatively interchangeable. Observed values depend on sensing frequency, electrode geometry, catheter contact, blood pool, wall thickness, and platform.
Fibrosis/heterogeneous border zone	Fibrotic remodeling alters conductivity, resistivity, permittivity, and phase angle through collagen deposition, loss of viable myocytes, altered extracellular volume, and disrupted cellular coupling. The direction and magnitude of these changes vary across experimental models and sensing frequencies [12,59,60,61,62,63,64,65,66].	LI demonstrates regional heterogeneity in fibrotic and infarct border-zone tissue. Intermediate-LI corridors and increased spatial LI heterogeneity have been associated with heterogeneous scar and critical VT substrate. The relationship between fibrosis and LI is additionally modified by catheter contact [25,37,38,67].	Heterogeneous border zones can support slow conduction and reentry and may contain critical channels relevant to VT or atrial substrate characterization.	LI may provide complementary information to voltage mapping, but no universal LI value defines fibrosis or border-zone tissue. Findings remain platform-, substrate-, and contact-dependent.
Acute edema/inflammation	Increased interstitial water and inflammatory cellular infiltration alter tissue conductivity and permittivity. These changes are dynamic and potentially reversible, in contrast to chronic scar remodeling [12,23].	Impedance measurements change after ablation as edema develops. Peri-procedural edema may transiently alter apparent lesion characteristics and can obscure residual viable tissue or incomplete injury [23,24].	Particularly relevant to assessment of acute ablation lesions and identification of potentially reversible conduction block or residual gaps.	Impedance changes caused by edema are not specific for irreversible necrosis. Measurements should therefore not be interpreted as direct indicators of durable lesion formation without complementary evidence.
Acute ischemia/early tissue injury	Acute ischemia produces cellular swelling and changes in intra- and extracellular water distribution, with reported increases in conductivity and permittivity in experimental studies [12,64].	Direct clinical evidence establishing a reproducible LI signature specific to acute myocardial ischemia is limited.	Potential biological basis for detecting evolving tissue injury or differentiating acute from chronic substrate.	Primarily mechanistic/preclinical evidence. A clinically validated, ischemia-specific LI threshold or dielectric signature has not been established.
Radiofrequency thermal injury/lesion formation	Heating alters conductivity and impedance through temperature-dependent changes in membrane integrity, protein denaturation, cellular injury, and tissue dehydration. Multi-frequency responses may differ between reversible and irreversible thermal injury [23,47,48].	Baseline LI, absolute LI drop, rate of LI decline, and temporal impedance dynamics correlate with acute lesion formation in several ablation studies [26,27,28,29,30,31,32,33,34,35,36]. LI responses also differ according to underlying tissue composition.	Provides real-time procedural information regarding catheter–tissue interaction and acute lesion formation during RF ablation.	LI thresholds are study- and platform-specific. Contact force, tissue type, wall thickness, power delivery, catheter configuration, and baseline impedance substantially affect the measured response.
Cellular membrane/T-tubular remodeling	Loss or disruption of intact cellular membranes and T-tubular architecture can alter membrane capacitance, intracellular conduction, and frequency-dependent dielectric behavior [22,57].	A specific, independently validated catheter-LI pattern for T-tubular loss has not been established in humans.	Provides a mechanistic explanation for how cellular remodeling could alter tissue electrical behavior and conduction.	Evidence is predominantly experimental. Clinical tissue classification based on these microscopic changes remains conceptual.

**Table 3:** Comparative characteristics of representative studies evaluating myocardial dielectric properties and local impedance. Data summarized from the individual studies cited in the first column. NR indicates information not reported in the source or not available in the reviewed text.

Study	Design/Model	Sample Size	Technique and Frequency	Tissue/ Arrhythmia	Main Findings	Principal Limitation	Clinical Significance
Amorós–Figueras et al. [59]	Chronic myocardial-infarction model	NR	Multi-frequency myocardial impedance; 1 and 41 kHz specifically reported	Normal, heterogeneous, and dense infarct scar	Resistivity decreased from normal to heterogeneous and dense scar; resistivity and phase angle correlated strongly with fibrosis	Experimental model; findings may not directly translate to human mapping	Supports impedance-based differentiation of scar density
Schwartzman et al. [60]	Experimental comparison of normal and chronically infarcted LV myocardium	NR	Multi-frequency impedance spectroscopy; 1 kHz values reported	Normal versus chronic infarct scar	Dense scar had lower resistivity and lacked the frequency-dependent capacitive response of normal myocardium	Older experimental study with limited applicability to current mapping systems	Provides foundational evidence that chronic scar has a distinct dielectric profile
DURABLE-I [24]	Human clinical study during PVI	NR	Wide-band dielectric imaging; exact acquisition frequencies NR	AF ablation lesions and residual conduction gaps	Gap identification was most informative before substantial edema; treatment of identified gaps reduced pulmonary vein reconnection from 95% to 25% at 1 month	Edema can mask incomplete necrosis; limited outcome data and platform-specific technology	Demonstrates potential for real-time differentiation of durable lesions from transient edema
Unger et al. [25]	Human atrial mapping study	NR	Local electrical impedance mapping; platform-specific frequency NR	Pulmonary veins, atrial myocardium, and prior ablation lesions	Pulmonary vein sleeves had higher LI than other atrial regions; previous ablation lesions showed lower LI than surrounding tissue	LI affected by anatomy, contact, and blood pool; observational design	Supports atrial tissue characterization and identification of prior lesions
Bates et al. [67]	Human observational study	NR	Local impedance with contact force assessment; frequency NR	Left-atrial tissue with differing fibrosis and rhythm	The relationship between contact force and LI differed between electrically active and fibrotic tissue	Fibrosis classification and catheter contact may confound one another	Suggests that tissue composition should be considered when interpreting LI
García–Bolao et al. [26]	Human repeat-PVI study with 3-month reassessment	NR	Local impedance mapping; frequency NR	Mature lesions, reconnected segments, and healthy atrial myocardium	LI differentiated mature ablated tissue, reconnected segments, and healthy myocardium; larger LI reductions were associated with durable block	Repeat-procedure population; thresholds may not generalize across platforms	Supports LI for identifying conduction gaps and lesion durability
Masuda et al. [27]	Human clinical PVI study	NR	Local impedance monitoring; frequency NR	AF ablation lesions	An LI drop of approximately 13.4 Ω predicted gap-free lesion formation with 78% sensitivity and 75% specificity	Acute and short-term endpoints; no universal threshold established	Provides a clinically usable but platform-dependent lesion-quality threshold
Garrott et al. [28]	In vitro and in vivo lesion-assessment study	NR	Combined LI and contact force sensing; frequency NR	RF ablation lesions	LI reduction correlated with lesion depth more strongly than generator impedance; combined parameters improved lesion assessment	Experimental conditions and tissue-contact effects limit direct clinical generalization	Supports combining LI with contact force rather than using either parameter alone
Osei et al. [29]	Preclinical standard- and high-power RF study	16 lesion sets reported for durability analysis	LI-guided RF ablation; frequency NR	Experimental atrial lesion formation	An LI target of 15–30 Ω produced durable conduction block in 16 of 16 lesion sets	Preclinical model; small number of lesion sets	Supports feasibility of LI-guided energy titration
Szegedi et al. [30]	Human pilot study during PVI	NR	Contact force catheter with LI monitoring; frequency NR	Anterior and posterior left-atrial walls	Optimal LI drop cutoffs differed by location: 21.8 Ω anteriorly and 18.3 Ω posteriorly	Pilot study; anatomical and wall-thickness dependence	Demonstrates that a single universal LI threshold is inappropriate
Perge et al. [31]	Human clinical study during PVI	NR	Temporal LI analysis during RF ablation; frequency NR	Acute atrial lesion efficacy	An LI drop of less than 9 Ω at 4 s predicted unsuccessful applications; successful lesions showed continued LI decline	Acute endpoint without long-term lesion validation	Shows that early LI kinetics may provide real-time feedback
Alken et al. [32]	Human repeat-AF-ablation study	NR	Combined contact force and LI dynamics; frequency NR	Reconnected and durable atrial lesions	Combined contact force and baseline LI predicted successful RF applications better than either parameter alone	Repeat-procedure population; moderate predictive performance	Supports multimodal procedural indices rather than isolated LI values
Aranyó et al. [37]	Post-infarction swine model	NR	Endocardial LI mapping; frequency NR	Scar-related VT substrate	Critical VT isthmuses localized to intermediate-LI corridors, including regions appearing homogeneous by voltage mapping	Animal model; selected ischemic substrate	Suggests that LI may reveal arrhythmogenic heterogeneity missed by voltage mapping
Aranyó et al. [38]	Translational chronic-infarction study with histology and CMR	NR	LI mapping correlated with histology and CMR; frequency NR	Dense scar and heterogeneous infarct border zone	Low LI corresponded to dense transmural scar; intermediate LI represented heterogeneous subendocardial scar; 75.6% of CMR border-zone corridors colocalized with intermediate LI	Limited sample and predominantly ischemic substrate	Provides biological and imaging validation for LI-defined tissue subtypes
Münkler et al. [39]	Human clinical VT ablation study	NR	LI-guided ventricular ablation; frequency NR	Healthy and scarred ventricular myocardium	Baseline LI was lower in scar; VT-terminating applications had larger and faster LI reductions than nonterminating applications	Small clinical study; no randomized comparison or long-term outcome validation	Supports LI as an adjunct during scar-related VT ablation
Duc et al. [47]	Ex vivo RF ablation experiments	NR	Multi-frequency myocardial bioimpedance spectroscopy	Thermal lesion formation in myocardial tissue	Impedance behavior varied with sensing frequency and temperature and may distinguish reversible from irreversible thermal changes	Ex vivo setting without clinical outcomes	Supports multi-frequency sensing for improved lesion characterization
Qian et al. [48]	Experimental lesion-formation study	NR	Multichannel impedance configuration	RF ablation lesions	Optimized impedance measurement correlated more closely with lesion dimensions than conventional generator impedance	Experimental study; specialized configuration and no outcome assessment	Demonstrates that electrode configuration materially influences lesion estimation
Takigawa et al. [49]	Ex vivo machine-learning study	1142 RF applications	Extreme gradient-boosting model using impedance change, energy, contact force, orientation, and duration	Excised ventricular myocardium and RF lesions	The machine-learning model estimated lesion dimensions more accurately than individual conventional indices	Ex vivo data; no arrhythmogenic-tissue classification or patient outcomes	Most direct evidence that machine learning can extract additional value from impedance-containing datasets
Bates et al. [68]	Human diseased-LV ablation study	NR	Local impedance during RF ablation; frequency NR	Normal, low-voltage, and dense scar ventricular tissue	Impedance responses to similar energy delivery differed according to underlying tissue composition	Tissue heterogeneity and observational design prevent a universal threshold	Reinforces the need for substrate-specific rather than universal LI targets

**Table 4:** Representative artificial intelligence and machine-learning systems relevant to cardiac electrophysiology and dielectric analysis.

System/Model	Primary Input	Analytical Approach	Output/Application	Validation Status	Direct Dielectric Input?
TAILORED-AF algorithm [43]	Intracardiac electrograms	Automated spatiotemporal-dispersion analysis	Identification of additional AF ablation targets	Randomized clinical study; 370 patients	No
Personalized cardiac digital twins [44,45,46]	LGE-CMR, cardiac anatomy, and electrophysiological data	Patient-specific computational simulation	Identification of VT substrate and potential ablation targets	Early prospective and feasibility studies	No
Takigawa et al. [49]	Ablation energy, duration, contact force, catheter orientation, and impedance change	Extreme gradient boosting	Estimation of RF lesion dimensions	Ex vivo; 1142 ablation applications	Yes—impedance included
DEEP RISK	LGE-MRI, 12-lead ECG, and clinical data	Residual variational autoencoders with multimodal machine learning	Prediction of malignant ventricular arrhythmias	Retrospective, two-center cohort of 289 patients; external validation AUROC 0.84	No
AI-ECG for LQTS	Twelve-lead ECG	Convolutional neural network	Detection of concealed LQTS and genotype prediction	Clinical diagnostic studies with internal or external validation	No
Proposed AI–dielectric framework	Raw dielectric/LI signals, contact data, electrograms, voltage maps, CT/CMR, and procedural variables	Multimodal machine learning with artifact correction and feature fusion	Tissue classification, gap detection, lesion durability, and outcome prediction	Conceptual; no prospective multicenter validation	Yes

## Data Availability

This review was based on publicly available academic literature databases.

## Abbreviations

AF, atrial fibrillation; AI, artificial intelligence; AMSA, amplitude spectral area; CMR, cardiac magnetic resonance; CT, computed tomography; ECG, electrocardiography; EIT, electrical impedance tomography; LI, local impedance; LGE, late gadolinium enhancement; LQTS, long-QT syndrome; PVI, pulmonary vein isolation; RF, radiofrequency; SVT, supraventricular tachycardia; TI, thoracic impedance; VF, ventricular fibrillation; VT, ventricular tachycardia.
